# Feasibility of multiplane microtransoesophageal echocardiographic guidance in structural heart disease transcatheter interventions in adults

**DOI:** 10.1007/s12471-017-1036-6

**Published:** 2017-09-08

**Authors:** V. J. Nijenhuis, A. Alipour, N. C. Wunderlich, B. J. W. M. Rensing, G. Gijsbers, J. M. ten Berg, M. J. Suttorp, L. V. A. Boersma, J. A. S. van der Heyden, M. J. Swaans

**Affiliations:** 1 Department of Cardiology, St Antonius Hospital, Nieuwegein, The Netherlands; 2 Department of Cardiology, Cardiovascular Centre Darmstadt, Darmstadt, Germany; 3 Philips Healthcare, Best, The Netherlands

**Keywords:** Transcatheter, Transoesophageal echocardiography (TEE), Intracardiac echocardiography (ICE), MicroTEE, Micro probe, Accuracy, Intervention

## Abstract

**Introduction:**

Structural heart interventions are guided by transoesophageal or intracardiac echocardiography (TEE/ICE). MicroTEE, developed for paediatric purposes, is smaller and therefore less invasive and traumatic, avoiding the need for general anaesthesia. We aimed to show feasibility of procedural guidance by comparing image quality of microTEE with standard TEE and ICE during adult transcatheter interventions, and assess the accuracy in obtaining left atrial appendage (LAA) measurements between the microTEE probe and standard TEE.

**Methods and results:**

We prospectively included 49 patients (20 women, 64 ± 18 years). Intraprocedural images were obtained by using the microTEE probe and standard (2D and 3D) TEE (LAA closure, MitraClip implantation) or ICE (interatrial communication closure, transseptal puncture for left atrial ablation). Two echocardiographers independently assessed image quality from 1 (excellent) to 4 (poor) and performed LAA measurements. Use of microTEE was not related to significant discomfort. Image quality obtained with the microTEE probe was lower than with standard TEE (2 [1–2] vs. 1 [1–2]; *p* = 0.04) and comparable with ICE images (2 [1–2] vs. 2 [1–2], *p* = 0.13). MicroTEE showed a wider field of view than ICE. LAA measurements on images obtained by microTEE were strongly associated with standard TEE.

**Conclusions:**

MicroTEE seems feasible for guidance during transcatheter heart interventions in adults. MicroTEE imaging offers a wider field of view than ICE, and its accuracy is comparable with TEE. In transcatheter interventions performed under conscious sedation, microTEE might be a viable and advantageous alternative to standard TEE or ICE.

## Introduction

Transoesophageal echocardiography (TEE) plays an essential role in the evaluation and monitoring of patients during cardiac interventions [1–5]. The standard two-dimensional (2D) TEE probe (S7-2 Omni TEE) measures 14.9 and 10 mm in tip and shaft diameter, whereas the 3D TEE probe (X7–2t) measures 16.6 mm by 9.5 mm. Recently, a new type of TEE probe has become available (S8–3t micro TEE probe, Philips Healthcare, Andover, MA, USA) which is currently the world’s smallest transducer for cardiac imaging of neonatal patients. This new probe allows multiplane 2D imaging and measures only 7.5 and 5.2 mm in tip and shaft diameter.

TEE using the standard size TEE transducer is usually well tolerated by the patient for a limited time. However, it carries the risk of inducing local trauma to the oropharynx, oesophagus and stomach [6]. The microTEE probe allows an easier, less traumatic transoesophageal passage, thereby potentially reducing discomfort and risk for these complications.

Transcatheter structural heart interventions are increasingly performed under local rather than general anaesthesia. When procedural guiding by microTEE proves sufficient, its use might lower the barrier to switch from general to local anaesthesia. This may result in shorter procedure times, fewer anaesthesia-related complications, more efficient planning and lower procedural costs.

However, since the microTEE probe contains fewer elements than standard TEE, image quality and accuracy might be suboptimal. In infants, the microTEE provides inferior image quality, but its accuracy is comparable with the paediatric multiplane 2D TEE probe [7]. Additionally, microTEE imaging demonstrated to be comparable with intracardiac echocardiographic (ICE) imaging for transseptal puncture guidance [8].

In the current study, we aimed to show feasibility in procedural guidance in terms of image quality rendered by the microTEE probe and standard TEE or ICE imaging during different transcatheter interventions in the treatment of structural heart diseases in adult patients. Additionally, we analysed the accuracy in obtaining left atrial appendage (LAA) measurements between the microTEE probe and standard TEE.

## Methods

### Design

**Fig. 1:**
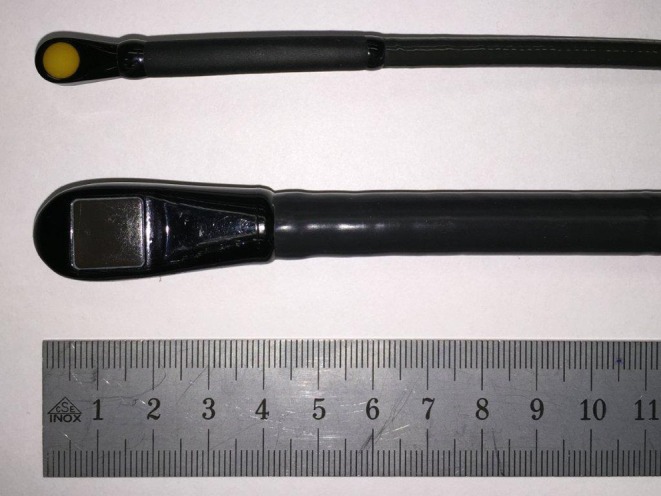
Transoesophageal probes. The S8–3t micro transoesophageal echocardiography probe (Philips Medical Systems, Andover, MA) (above) and the 3DTEE probe (X7–2t, Philips Medical Systems)

This was a prospective, observational unblinded study to determine the capabilities of microTEE probe imaging to guide the operator during interventional therapy in cardiac disease patients (Fig. 1).

### Objectives

The primary objective of this clinical study was to establish that microTEE imaging is able to visualise required anatomical features with similar image quality compared with TEE and ICE to ensure its feasibility in procedural guidance. Accuracy was tested on standard measurements of the LAA and compared between microTEE and TEE. The secondary objective was to establish that microTEE can be used without the use of general anaesthesia.

### Patient selection

The study was performed on patients undergoing either one of the following cardiac interventions between October 2014 and November 2015: (i) LAA closure, (ii) interatrial communication closure, and (iii) atrial transseptal puncture for other purposes.

Patients were included if they fulfilled the following inclusion criteria: (i) subject is 18 years of age or older, and (ii) has given written informed consent to participate in the study. No patients were excluded. This study was approved by a medical ethics committee (NL49744.100.14).

### Study procedures

Intraprocedural images of the LAA were recorded with microTEE and subsequently (i) standard 2D/3D TEE in patients undergoing LAA closure and a transseptal puncture for MitraClip implantation, and; (ii) ICE in patients undergoing interatrial communication closure and atrial transseptal puncture during left-sided ablations such as pulmonary vein isolation. The ICE catheter was inserted into the inferior vena cava and positioned within the right atrium. To reduce the bias of prior knowledge of anatomy, microTEE was used prior to TEE or ICE in all patients.

After anaesthesia was induced, the microTEE probe was introduced either transnasally (initial 10 patients) or transorally into the oesophagus (subsequent patients). A full set of images was obtained using standard views. Hereafter, images with standard size 2D/3D TEE probe or ICE were performed. To evaluate patient discomfort during the placement of the microTEE probe, patients undergoing local anaesthesia were asked to evaluate their comfort on a visual analogue scale from 0 (no discomfort or pain) to 10 (unbearable pain).

**Fig. 2:**
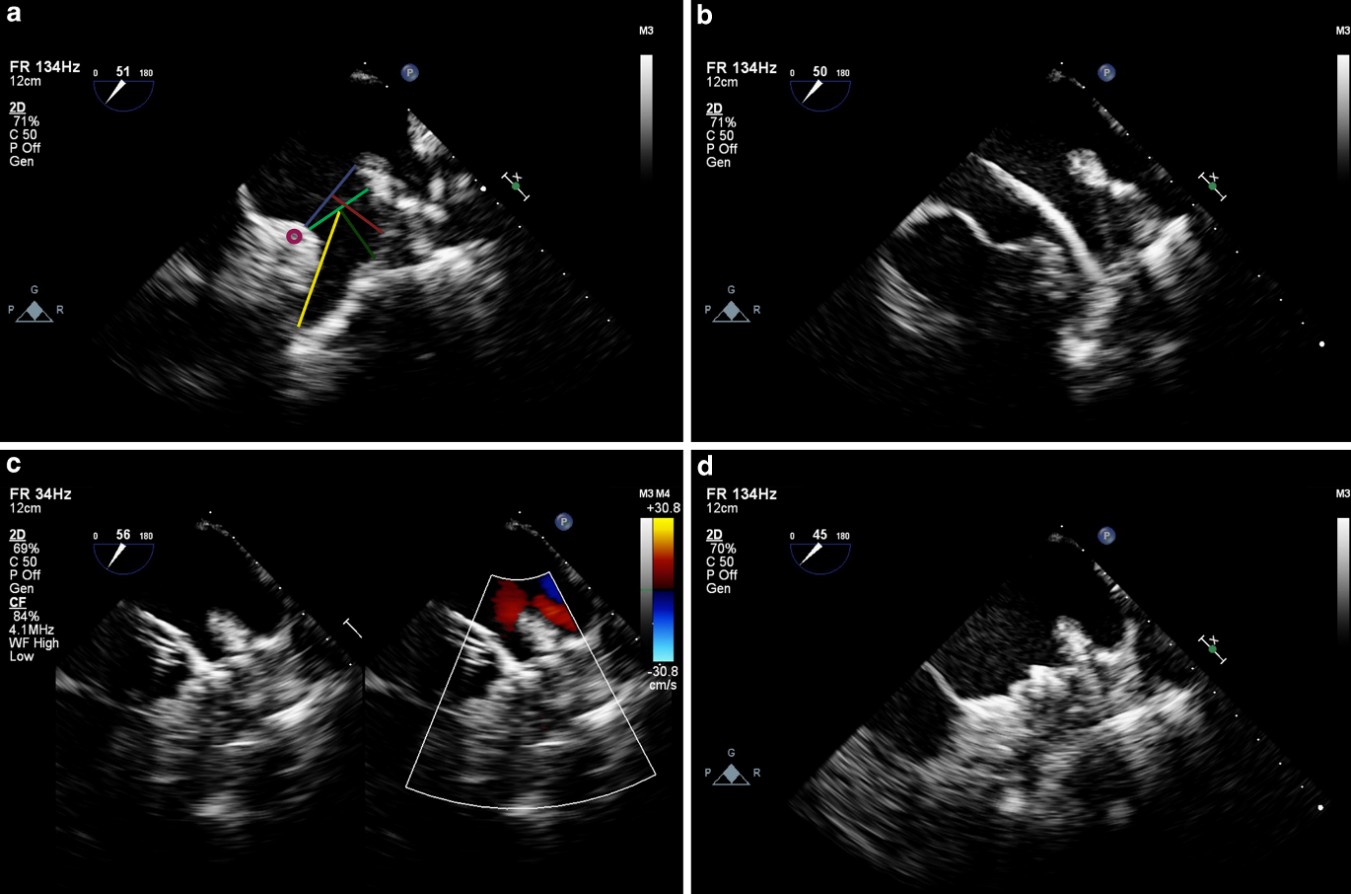
Microtransoesophageal echocardiographic images during left atrial appendage closure. Microtransoesophageal echocardiography during left atrial appendage (LAA) closure. **a** The LAA is shown at 51 degrees, and the dimensions routinely measured for LAA closure are drawn: ostium (blue), landing zone (light green), depth of ostium (red), depth of landing zone (dark green), and the total depth (yellow). **b** A Watchman device is advanced in the LAA. **c** The Watchman device is deployed and colour compare mode shows no residual flow. **d** The Watchman device is released

Two experienced echocardiographers (M.J.S., V.J.N.) independently evaluated each recording in random order. To assess image quality, each echocardiographer assigned an image quality score of 1 to 4 (1 = excellent; 2 = good; 3 = fair; 4 = poor) describing how well structures were seen on the particular recording. Additionally, to assess accuracy, each echocardiographer independently performed measurements of the LAA for appropriate device sizing [1, 9] as depicted in Fig. 2. These measurements included diameters of the ostium, landing zone, depth from the ostium needed for Amplatzer (AGA, St. Jude Medical, Minneapolis, MN, USA) closure, depth from the landing zone needed for the Watchman (Atritech, Boston Scientific, Natick, MA, USA) closure, and the total depth of the LAA. Each structure was measured at the following views: 0°, 45°, 90°, and 135°. Atrial septal defect measurements were not performed.

### Study device

The S8–3t microTEE probe (Philips Healthcare) has a shaft width of 5.2 mm and length of 85 mm, a transducer tip width of 7.5 mm, height of 5.5 mm, and length of 18.5 mm (Fig. 1). The microTEE transducer is a 32-element phased array transducer that is equipped with 2D B‑mode echocardiography, colour Doppler, pulse-wave and continuous-wave Doppler, M‑mode, and colour flow M‑mode features. It has a centre frequency of 6 MHz on a bandwidth of 3.2 to 7.4 MHz.

The X7–2t 3D TEE-probe (Philips Healthcare), which has an imaging frequency range from 7 to 2 MHz, was used as the standard 2D/2D TEE probe (Fig. 1). Its head has a width, height and length of 16 × 12 × 40 mm. The shaft is 10 mm in diameter and 106 cm long. The probe contains >2,500 elements.

Both the standard 2D/3D TEE and microTEE probe provide a 180° manual image plane control with angular display, anterior and posterior articulation and an articulation brake. Both probe tip surfaces are constantly monitored for temperature to ensure patient safety.

For ICE, the Acuson AcuNav catheter (Siemens Medical Solutions USA, Inc., Mountain View, CA) was used: an 8 French, 2.5 mm, 64-element, linear phased array transducer that is equipped with a single longitudinal plane. Its imaging frequency ranges from 5.5 to 10 MHz.

### Statistical analysis

The image quality scores [1–4] and the measured LAA dimensions (mm) were presented as median (interquartile range) and mean ± standard deviation (SD), respectively. The measured diameters were provided as the mean of all four standard views by both echocardiographic reviewers. The results of the microTEE were compared with the results of the standard 2D/3D TEE probe and ICE using a paired samples t‑test and dependent Wilcoxon signed-rank test, as appropriate. Accuracy was tested on pre-specified LAA measurements, because these measurements are validated and consider a standardised and reproducible set, by using Pearson’s r correlation test and mean difference according to the Bland-Altman plot. The two-way mixed intraclass correlation coefficient was used to assess interobserver variability (M.J.S. and V.J.N.). Statistical significance was inferred at *p* < 0.05.

## Results

### Baseline characteristics

In total, 49 patients were included. There were 20 (49%) women and 29 (51%) men, with a mean age of 64 ± 18 years. Patients underwent the following interventions: LAA closure (*n* = 18, 37%); interatrial communication closure (*n* = 14, 29%); and transseptal puncture for MitraClip implantation (*n* = 12, 24%) or left-sided ablation (*n* = 5, 10%). General anaesthesia was used in 27 (55%) procedures. In those undergoing local anaesthesia, all patients had no to mild discomfort; 1 patient (5%) had moderate discomfort regarding the microTEE. In the first 10 (20%) patients, the microTEE was introduced transnasally. In one of these cases, minimal epistaxis occurred after which we decided to move to transoral introduction.

### Qualitative assessment

Image quality scores are provided in Tab. 1 and 2. There was no qualitative difference in the ease of passage and ability to manipulate the microTEE compared with the standard TEE probe. The standard probe rendered a better overall average quality score than the microTEE probe (1 [1, 2] vs. 2 [1–2]; *p* = 0.04). Although the overall average scores were significantly different, both scores were between good (2) and excellent (1) in our rating, and good visualisation was achieved using both probes. No anatomical features were missed by the microTEE. Compared with ICE imaging, the microTEE probe offered comparable image quality (2 [1–2] vs. 2 [1–2]; *p* = 0.13) with a marked increased field of view.

**Table 1:** Image quality: MicroTEE imaging compared with standard TEE imaging

	MicroTEE	Standard TEE	*p*-value
LAA during LAAC (*n* = 18)	2 (1.25–2)	1 (1–2)	0.01
TSP during MitraClipping (*n* = 12)	2 (1–2)	2 (1–2)	0.75
Total (*n* = 30)	2 (1–2)	1 (1–2)	0.04

Results are provided in mean ± standard deviation; *LAA* left atrial appendage, *LAAC* left atrial appendage closure, *TSP* interatrial transseptal puncture

**Table 2:** Image quality compared with ICE

	MicroTEE	ICE	*p*-value
ICC (*n* = 14)	2 (2–2)	1.5 (1–2)	0.35
TSP (*n* = 5)	2 (1.75–2.25)	2 (1–2)	0.18
Total (*n* = 19)	2 (1–2)	2 (1–2)	0.13

Results are provided in mean ± standard deviation; *ICC* interatrial communication closure, *TSP* interatrial transseptal puncture

### Accuracy

LAA measurements are provided in Tab. 3. Compared with standard 2D/3D TEE imaging, measurements seemed to be underestimated when the microTEE probe was used (Tab. 3). Bland-Altman plotting revealed a mean difference of −1.2 mm (95% CI −2.4 to 1.7 mm) for the ostium, −0.9 mm (95% CI −2.1 to 2.1 mm) for the landing zone, −0.9 mm (95% CI −2.4 to 3.0 mm) for the ostium’s depth, −1.3 mm (95% CI −3.4 to 0.9 mm) for the landing zone’s depth. The total depth of the LAA did not differ. The Pearson correlation coefficients between microTEE and standard TEE were *r* = 0.95 (*p* < 0.01) for the ostium, *r* = 0.94 (*p* < 0.01) for the landing zone, *r* = 0.88 (*p* < 0.01) for the depth of ostium, *r* = 0.95 (*p* < 0.01) for the depth of the landing zone, and *r* = 0.93 (*p* < 0.01) for the total depth. The intraclass correlation coefficient for interobserver variability was 0.92 (*p* < 0.01) for the ostium, 0.89 (*p* < 0.01) for the landing zone, 0.90 (*p* < 0.01) for the depth of ostium, 0.90 (*p* < 0.01) for the depth of landing zone, and 0.95 (*p* < 0.01) for the total depth.

**Table 3:** Accuracy during LAA closure (*n* = 18)

	MicroTEE	Standard TEE	*p*-value
Ostium (mm)	20 ± 4	22 ± 3	<0.01
Landing zone (mm)	18 ± 4	18 ± 3	0.02
Depth of ostium (mm)	15 ± 3	16 ± 3	0.02
Depth of landing zone (mm)	15 ± 4	16 ± 3	<0.01
Total depth (mm)	26 ± 7	26 ± 6	0.53

Results are provided in mean ± standard deviation; *TEE* transoesophageal echocardiography. Measurement sites are specified in Fig. 2

## Discussion

Echocardiographic imaging guidance remains indispensable for a safe and accurate execution of transcatheter structural heart interventions [5]. TEE is preferred in the guidance of LAA closure and transcatheter valve interventions, whereas ICE is an alternative imaging option particularly in the guidance of interatrial communication closure and transseptal puncture [10, 11]. Nowadays, a large number of these procedures are performed outside the operating theatre under local rather than general anaesthesia. For these cases, less invasive echocardiographic imaging using the microTEE rather than standard TEE or ICE may prove beneficial. Therefore, we compared image quality by the microTEE probe and standard sized TEE probe as well as ICE. Accuracy between the microTEE probe and standard sized TEE probe was assessed regarding LAA measurements.

Procedures with general anaesthesia include the risk of hypotensive periods, hypotonia of the hypopharyngeal muscles, obstructive sleep apnoea, longer procedure times, and higher personnel costs [12–14]. General anaesthesia can be avoided by using ICE, but this imaging modality may also be suboptimal as it requires central vein cannulation as well as a great level of experience with a significant learning curve. Other limitations of ICE are interference by other catheters sharing the entrance of the inferior vena cava, a limited field of view, and potentially increased cost (Tab. 4).

**Table 4:** Advantages and limitations of standard TEE, ICE, microTEE and ClariTEE

Imaging modality	2D	3D	2D Image resolution	Invasive	General anaesthesia	Local anaesthesia	Costs
Standard TEE	+	+	+++	++	+ (In most centres)	− (In most centres)	−
ICE	+	(+)	+++ (Limited penetrating frequencies)	+++ (Central vein cannulation)	−	+	+ (Single use)
MicroTEE (S8–3 t, Philips Healthcare)	+	−	++	+	−	+	−
ClariTEE (ImaCor, Inc.)	+	−	++	+	−	+	+ (Single use)

To overcome these limitations, the microTEE system (S8–3t, Philips Healthcare) might be an attractive alternative. This probe provided good quality images in very small infants [15]. Additionally, it could be demonstrated that the intraoperative and postoperative use of the microTEE probe provides adequate accuracy albeit inferior image quality when compared with the paediatric multiplane TEE probe in infants [7, 16]. An alternative to the microTEE might be the disposable ClariTEE catheter (Imacor, Inc., New York, USA). However, compared with microTEE, the costs are increased due to its disposable character (Tab. 4).

Our study shows that standard TEE provides a better image quality than microTEE. This was expected, as the microTEE probe has a smaller number of elements and consequently a lower spatial resolution. Furthermore, the microTEE probe does not fill the oesophagus equally and may suffer from less optimal acoustic coupling with the heart. However, microTEE imaging provides sufficient image quality for procedural guidance as shown in Fig. 2.

**Fig. 3:**
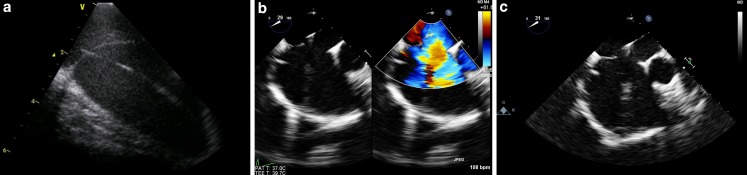
Intracardiac echocardiography compared with microtransoesophageal echocardiographic images. Intraprocedural images during atrial septum defect (ASD) II closure in an adult patient. **a** Intracardiac echocardiography (ICE) showing the intra-atrial septum and the catheter. **b** Microtransoesophageal echocardiography (TEE) colour compare mode showing the ASD (left) and the flow through the ASD (right) in a short axis at the base view. **c** MicroTEE short axis at the base view, showing a much wider field of view (visibility of the aorta) compared with ICE in (**a**)

During closure of interatrial communications, the microTEE probe provided comparable image quality to ICE. However, microTEE provided a much wider field of view showing more of the surrounding structures, which is beneficial for the procedural guidance (Fig. 3). An example of a percutaneous atrial septal defect closure procedure under microTEE guidance is shown in Fig. 3, demonstrating the wider field of view.

MicroTEE showed an adequate accuracy compared with standard TEE. MicroTEE-acquired measurements of the LAA showed an excellent correlation with standard TEE but tended to be underestimated. Besides measurements of the LAA, accuracy was not further evaluated. Although we encountered no device undersizing in patients undergoing LAA closure, this small underestimation with microTEE should be taken into account when choosing the LAA occluder size.

In the current study, we did not assess microTEE during transcatheter valve interventions. Given the lack of 3D imaging and a reduced image quality of deeper localised cardiac structures, microTEE may perform less optimally in these kind of interventions but further studies are needed.

## Conclusion

During transcatheter heart interventions in adults, microTEE is feasible for procedural guidance. It offers comparable image quality with a much wider field of view than ICE, and a slightly reduced but sufficient image quality and comparable accuracy as standard TEE. MicroTEE is not associated with any significant discomfort in patients under local anaesthesia. Our findings support the use of microTEE as a valid alternative imaging option compared with standard TEE or ICE. Further studies are warranted to assess other important potential benefits of the microTEE, such as shorter procedure times, fewer complications, a more efficient procedural planning and lower medical costs.
